# Late-Onset Manifestations of Von Hippel-Lindau Syndrome: A Case Report

**DOI:** 10.7759/cureus.62756

**Published:** 2024-06-20

**Authors:** Tushar Kalekar, Sai Pavan Kumar, Apurvaa Pachva

**Affiliations:** 1 Radiology, Dr. D. Y. Patil Medical College, Hospital and Research Centre, Dr. D. Y. Patil Vidyapeeth, Pune (Deemed to be University), Pune, IND

**Keywords:** von hippel-lindau syndrome (vhl), ultrasonography (usg), ct (computed tomography) imaging, mri imaging, cerebellar hemangioblastoma, adrenal pheochromocytoma

## Abstract

Von Hippel-Lindau (VHL) syndrome is characterized by a range of tumors including phaeochromocytomas, pancreatic adenomas, cerebellar haemangioblastomas, and renal cell carcinomas. A 50-year-old male presented with a three-week history of headache. Additionally, the patient exhibited signs of hypertension. Ultrasonography (USG) abdomen and pelvis showed a solid mass lesion in the left adrenal gland, iso-echoic to the renal cortex. On contrast-enhanced computed tomography (CECT) of the brain, a well-defined solid cystic lesion was seen in the left posterior cerebellar hemisphere. Small nodular enhancing lesions were seen in the right cerebellar hemisphere. On further imaging with MRI brain contrast, the lesions in the cerebellum were diagnosed as multifocal hemangioblastomas. Laboratory investigations revealed elevated urinary metanephrines and normetanephrine, suggesting pheochromocytoma. Based on radiological and biochemical investigations, with the features of cerebellar haemangioblastomas and pheochromocytoma, a diagnosis of VHL syndrome was made.

## Introduction

Von Hippel-Lindau (VHL) syndrome is inherited as an autosomal dominant gene. It occurs in 1 out of every 39,000 live births [1]. Mutations in the VHL gene may be passed down through generations and cause VHL [2,3]. The severity of this condition varies from person to person, but by the time a person reaches the age of 65, more than 90% of the affected individuals will have symptoms [4]. Certain abnormalities affecting the central nervous system (CNS) and the internal organs are seen in patients with VHL [5,6]. Neoplasms, such as hemangioblastomas and endolymphatic sac tumors, may form in the CNS. Respiratory cysts, pancreatic cysts, phaeochromocytomas, and cystadenomas of the adnexal organs (the broad ligament in women and the epididymis in men) are all possible visceral tumors. Patients with VHL had a median survival of 50 years until widespread monitoring and clearer guidelines for treating lesions associated with the disease became available [6,7]. Neurological hemangioblastoma and renal cell carcinoma (RCC) account for most deaths in VHL patients.

## Case presentation

A 50-year-old man presented with a three-week history of intermittent episodes of holo cranial headache associated with nausea and dizziness and was admitted to the hospital as the symptoms aggravated for three days. The patient’s blood pressure was elevated to 220/110 mmHg, with all other vitals within normal ranges. The remainder of the physical examination was unremarkable. There was no significant family history. The patient had a normal diet and sleep, normal urine and defecation, no significant weight loss, and no other discomforts.

Ultrasonography (USG) of the abdomen and pelvis was performed using a Samsung HS-70A ultrasonograph (Samsung, Seoul, South Korea). Contrast-enhanced computed tomography (CECT) of the brain was done using a Philips Ingenuity 128-slice CT scanner (Philips Healthcare, Amsterdam, Netherlands). Magnetic resonance imaging (MRI) of the brain with Gadolinium contrast was done using the Siemens 3 Tesla Magnetom VIDA MRI (Siemens Healthineers, Erlangen, Germany).

**Figure 1:**
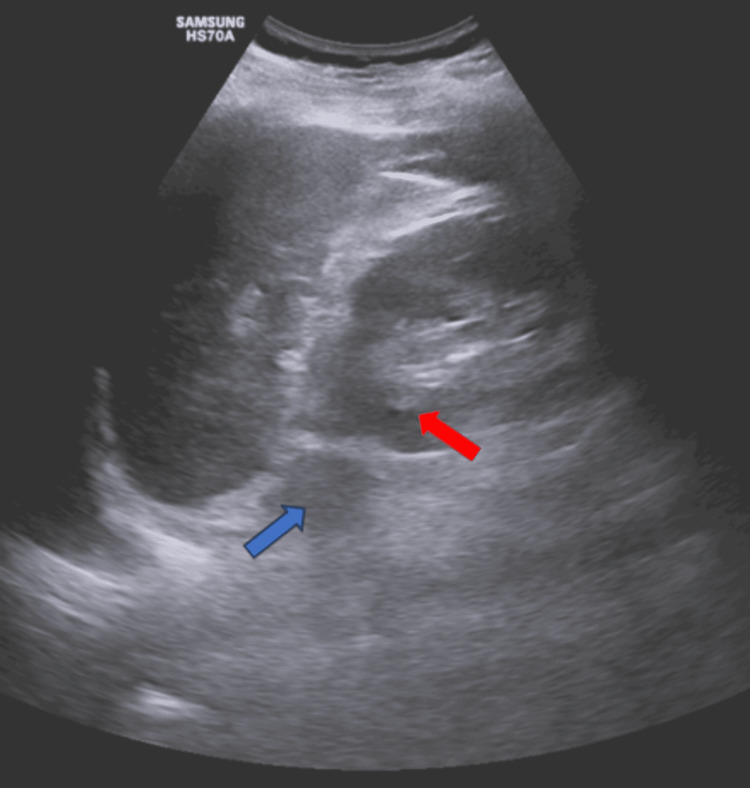
Ultrasonography of the abdomen and pelvis This figure shows a solid mass lesion in the left adrenal gland (blue arrow), which is iso-echoic to the left renal cortex (red arrow)

USG of the abdomen and pelvis showed a solid mass lesion in the left adrenal gland (Figure 1), which is isoechoic to the renal cortex.

**Figure 2:**
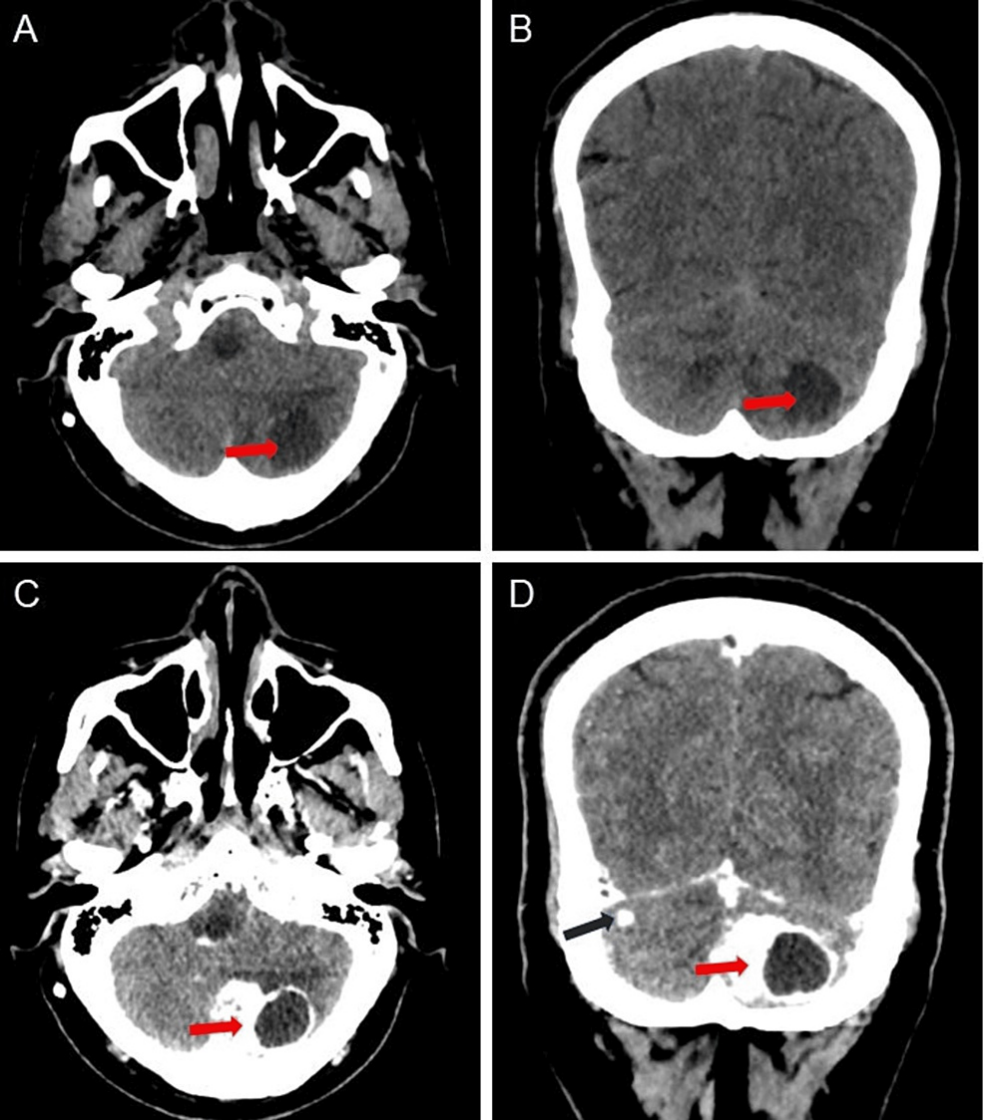
Axial and coronal reformatted non-contrast and post-contrast computed tomography images of the brain (A)-(B): Axial and coronal reformatted non-contrast computed tomography images show a solid cystic lesion in the left posterior cerebellar hemisphere (red arrows) (C)-(D): Axial and coronal reformatted post-contrast images show avid post-contrast enhancement of solid mural nodules (red arrows), and a small enhancing lesion is seen in the right cerebellar hemisphere (black arrow)

Non-contrast computed tomography of the brain was done, which revealed a well-defined solid cystic lesion in the left posterior cerebellar hemisphere (Figures 2A-2B). On post-contrast administration, the solid mural nodule shows avid post-contrast enhancement with a few tiny non-enhancing areas within - likely necrotic areas (Figures 2C-2D). The cystic component at the lateral aspect of the mural nodule showed peripherally enhanced walls in the contrast study. A small, enhancing lesion is seen in the right cerebellar hemisphere (Figure 2D). Surrounding hypodense vasogenic edema is seen, causing mild compression over the fourth ventricle, cerebral aqueduct, and quadrigeminal cistern.

**Figure 3:**
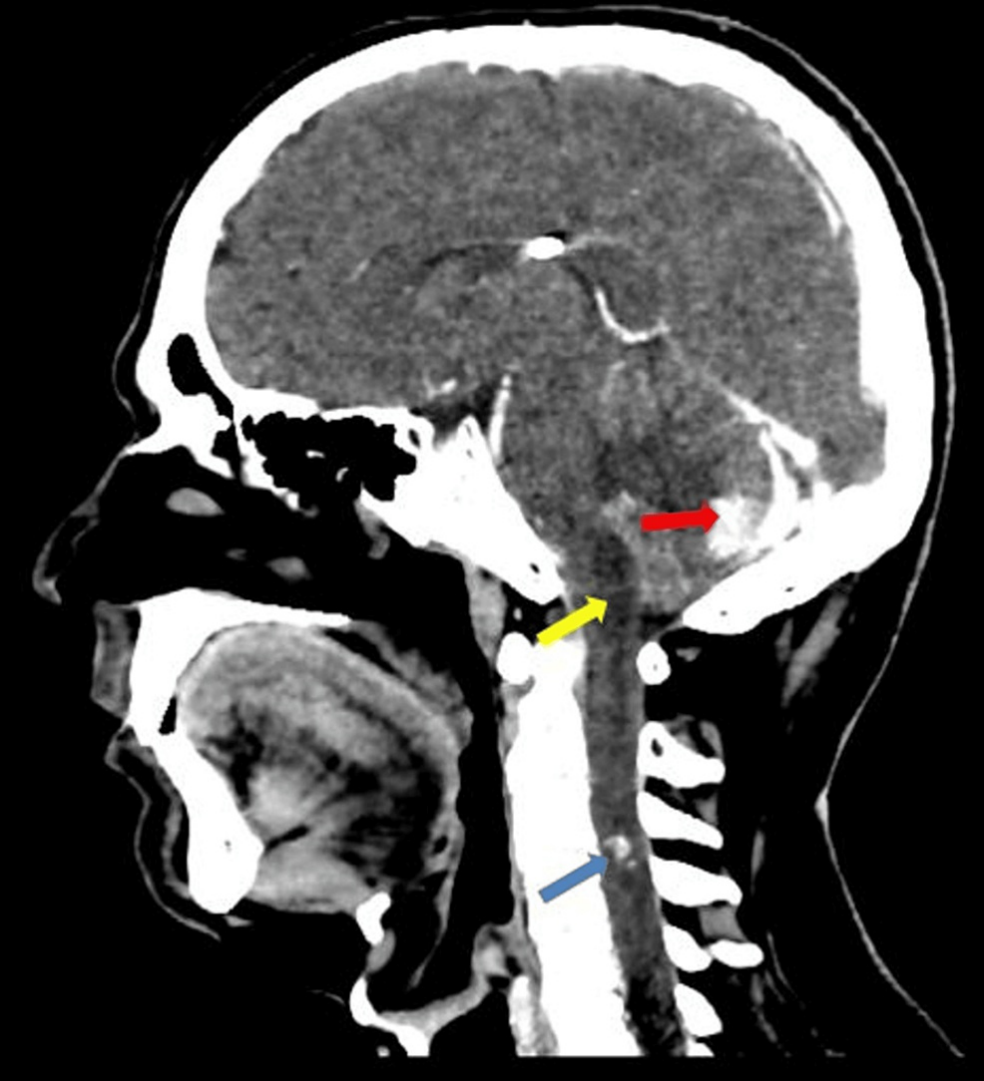
Sagittal reformatted contrast-enhanced computed tomography image This figure shows an enhancing lesion in the cervical spinal cord (blue arrow), with the central canal of the spinal cord above exhibiting hydromelia with a syrinx (yellow arrow), and an enhancing lesion in the left cerebellar hemisphere (red arrow)

Enhancing lesions are also noted in the cervical portion of the spinal cord (Figure 3). The central canal of the spinal cord showed CSF density with no post-contrast enhancement, extending from the cervico-medullary junction to the C4 vertebra, suggestive of hydromelia, possibly with a syrinx (Figure 3). Based on the above features, possible differentials of pilocytic astrocytoma with metastases or hemangioblastomas were given. Further evaluation with an MRI brain contrast was done.

**Figure 4:**
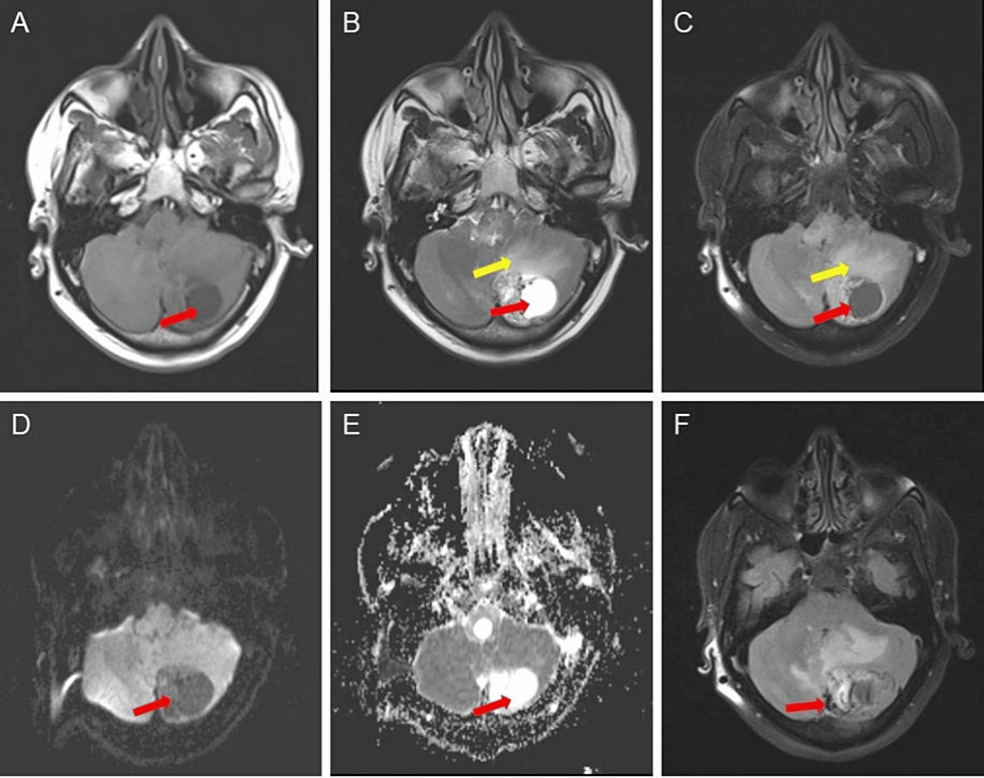
Multi-sequence MRI findings of a lesion in the left cerebellar hemisphere, demonstrating varied signal characteristics and associated features A) Axial T1 MRI image showing a hypointense solid-cystic lesion in the left cerebellar hemisphere (red arrow); B) T2 axial MRI image showing a hyperintense cystic component (red arrow) with T2 hyperintense vasogenic edema (yellow arrow); C) FLAIR axial image showing a hypointense lesion in the left cerebellar hemisphere (red arrow) with surrounding FLAIR hyperintense vasogenic edema (yellow arrow); D) DWI image showing no areas of diffusion restriction in the lesion; E) ADC map showing a bright signal in the lesion (red arrow); F) GRE sequence showing a focus of blooming suggesting hemorrhage or calcification (red arrow) FLAIR: Fluid attenuated inversion recovery; DWI: Diffusion-weighted imaging; ADC: Apparent diffusion coefficient; GRE: Gradient recalled echo

MRI revealed a well-defined, solid cystic lesion appearing as T1 hypointense (Figure 4A) and T2 hyperintense (Figure 4B), and showing suppression on the fluid-attenuated inversion recovery (FLAIR) sequence (Figure 4C). Serpentine flow voids of vessels are seen overlying the lesion. The peripheral mural nodule appears isointense on T1 and hyperintense on T2, shows no diffusion restriction on diffusion-weighted imaging (DWI) (Figure 4D), and exhibits a high apparent diffusion coefficient (ADC) value (Figure 4E). A focus of blooming was noted on the gradient recalled echo sequence (GRE) in the mural nodule (Figure 4F), suggesting hemorrhage or calcification. Surrounding T2/FLAIR hyperintense vasogenic edema was noted (Figures 2B-2C).

**Figure 5:**
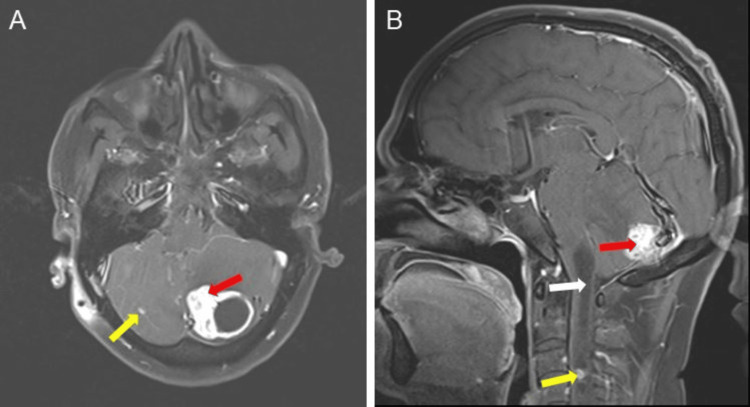
Multi-sequence MRI findings illustrating lesions in the brain and spinal cord A) MRI axial T1 post-contrast fat-saturated image shows intense contrast enhancement of the mural nodule (red arrow) with peripheral enhancing walls. A small, enhancing lesion is seen in the right cerebellar hemisphere (yellow arrow) B) Sagittal T1 post-contrast fat-saturated image shows fluid density in the cervical spinal cord from the cervico-medullary junction to the C4 vertebra, suggesting hydromelia with a syrinx (white arrow). An enhancing lesion is noted in the cervical spinal cord (yellow arrow). Left cerebellar lesion showing post-contrast enhancement (red arrow)

The mural nodule shows avid post-contrast enhancement with peripheral enhancing walls (Figure 5A). A small enhancing lesion is seen in the right cerebellar hemisphere (Figure 5A). Enhancing lesions are also noted in the cervical portion of the spinal cord (Figure 5B). The spinal cord in its central part shows fluid density with no post-contrast enhancement, extending from the cervico-medullary junction to the C4 vertebra, suggestive of syrinx (Figure 5B).

**Figure 6:**
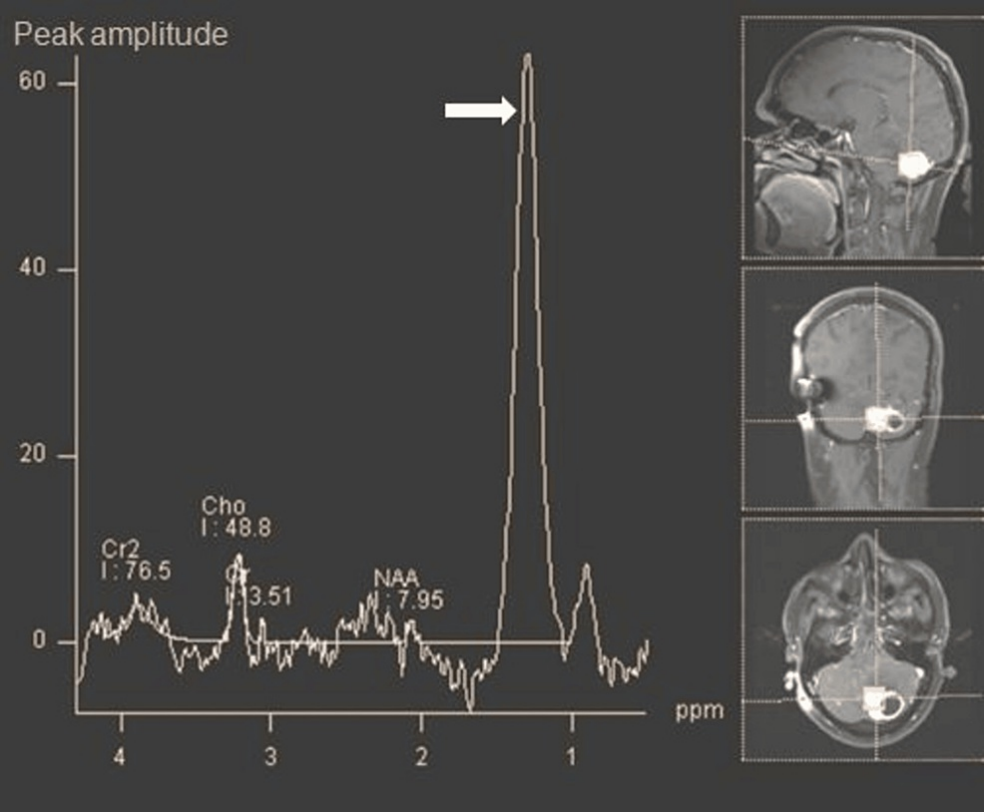
MR spectroscopy within the mural nodule This figure reveals a significant lipid/lactate peak (white arrow), the elevation of choline (cho), and a reduction in N-acetyl aspartate (NAA), suggesting hemangioblastoma

MR spectroscopy within the mural nodule reveals a significant lipid/lactate peak, elevation of choline, and reduction in N-acetyl aspartate (NAA) (Figure 6). MRI features mostly represented multifocal intracranial and spinal hemangioblastomas. Laboratory investigations revealed elevated urinary metanephrines and normetanephrine.

**Figure 7:**
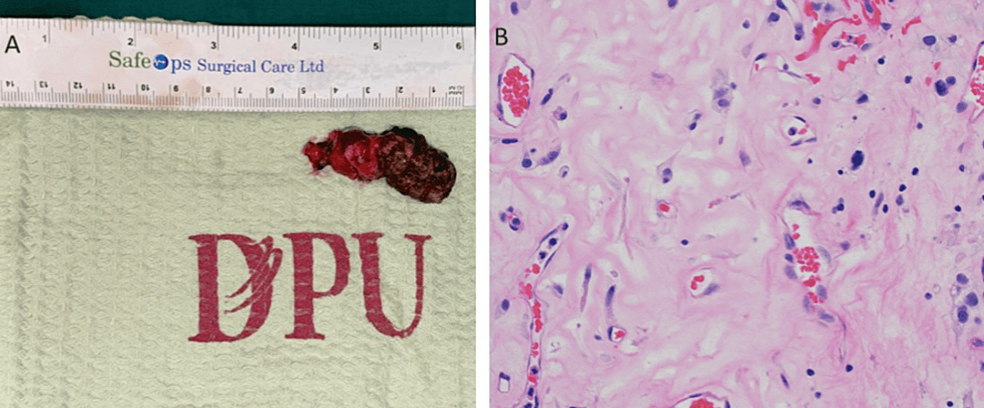
Excision of the cerebellar tumor and H&E-stained slide from the tumor resection A) Specimen of excised cerebellar tumor B) The hematoxylin and eosin (H&E) stained slide from tumor resection showed capillary channels with stromal cells and foamy cells with clear cytoplasm, diagnostic of hemangioblastoma (H&E, x400)

Excision of the cerebellar tumor was done (Figure 7A). The hematoxylin and eosin (H&E) stained slide from tumor resection showed capillary channels with stromal cells and foamy cells with clear cytoplasm, diagnostic of hemangioblastoma (Figure 7B).

**Figure 8:**
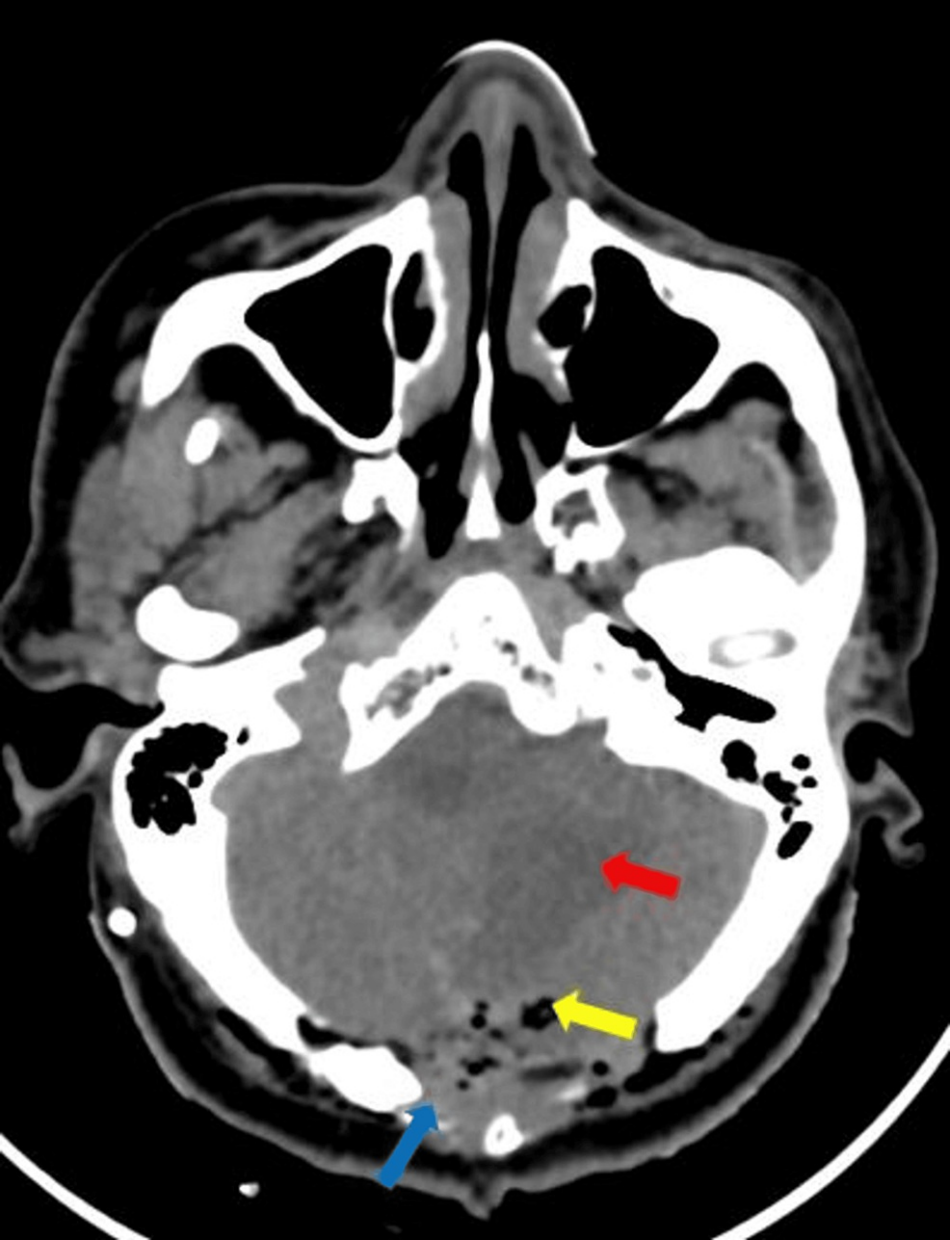
Post-operative non-contrast computed tomography This figure shows a craniotomy defect (blue arrow) in the occipital region with multiple air foci and hemorrhagic foci (yellow arrow) underneath the site of the defect, mostly postoperative changes. Hypodensity and edema are also seen in the right cerebellar hemisphere (red arrow)

Post-operative non-contrast computed tomography was performed. Craniotomy defect seen in the occipital region with multiple air foci and hemorrhagic foci seen underneath the site of the defect, mostly postoperative changes. Hypodensity and edema are seen in the right cerebellar hemisphere (Figure 8). After two months of follow-up, the patient's neurological symptoms were relieved and relatively stable.

## Discussion

Clinical criteria or genetic testing may confirm a diagnosis of VHL. For a patient to be diagnosed with VHL, they must have a history of the disease in their family as well as a CNS hemangioblastoma (including retinal hemangioblastomas), RCC, pheochromocytoma, or endothelial lining tumor. Two or more CNS hemangioblastomas, or one CNS hemangioblastoma plus a visceral tumor associated with VHL, will meet the clinical diagnostic criteria for VHL in about 20% of individuals without a family history of the disease [7,8].

In VHL, pheochromocytomas may be bilateral or numerous. The carotid body, glomus jugulare, and peri-aortic tissues may also host these tumors as extra-adrenal paragangliomas. According to research, 5% of pheochromocytomas are cancerous [9]. Although symptoms, such as headaches, pallor, nausea, tachycardia, palpitations, episodic sweating, and intermittent or chronic hypertension, are common in pheochromocytomas, 30% of patients may have no symptoms at all [9]. Imaging and laboratory tests confirm a pheochromocytoma diagnosis. In order to avoid a perioperative hypertensive crisis, it is essential to assess VHL patients before surgery. Through early surgical intervention and adrenal cortical-sparing surgery, patients are able to achieve long-term independence from corticosteroids with little recurrence.

A CNS hemangioblastoma of the cerebellum, brainstem, or spinal cord will develop in 60-80% of VHL patients throughout the course of their lifespan [5]. Several hemangioblastomas will occur in almost all of these individuals (around 90%). Hemangioblastomas are benign on histology, but they may cause serious illness and even death in VHL. The brainstem, cerebellum, or spinal cord account for virtually all cases of CNS hemangioblastomas (tumors outside the retina) (95% of tumors) [5,10,11]. Depending on the specific location of the tumor, patients with CNS hemangioblastomas may have a variety of symptoms. Headaches (75%), ataxia of gait (55%), dysmetria (29%), hydrocephalus (28%), and nausea/vomiting (28% of patients) are symptoms of cerebellar hemangioblastomas [12]. For the detection and monitoring of changes in hemangioblastomas, the most sensitive and reliable imaging technique is contrast-enhanced craniospinal MR imaging.

In a study that followed 19 patients with VHL for at least 10 years, Ammerman et al. [13] examined the trend of the development of CNS hemangioblastomas by serial imaging. In virtually all cases (97% of 143 tumors), hemangioblastomas exhibited a saltatory development pattern with bursts of fast growth interspersed with periods of relative quiet. The average number of quiescent periods between growth stages for hemangioblastomas was 1.85 before they became symptomatic and needed to be removed. On average, there were 13±15 months of growth phases and 25±19 months of growth quiescence periods. Radiographic progression was seen in almost all the hemangioblastomas (97%); however, only 50% of those tumors needed therapy to cause symptoms.

Most craniospinal hemangioblastomas may be safely removed, and hemangioblastoma complete resection is curative [12,14,15]. Patients will usually regain their pre-treatment neurologic condition within six weeks after CNS hemangioblastoma excision, and modest, transitory impairments in neurologic function are rare [5,12]. Surgery is often postponed until the onset of related symptoms occurs due to the multiplicity and unpredictable development rate of CNS hemangioblastomas [11,14]. This approach allows for the avoidance of needless surgical resection and the preservation of good neurologic function in the majority of VHL patients [14].

## Conclusions

Multifocal intracranial and spinal hemangioblastomas pose diagnostic challenges due to their rare occurrence and diverse clinical presentations. Through meticulous imaging evaluation, including USG, CT, and MRI, coupled with advanced techniques like MR spectroscopy, we were able to accurately diagnose and delineate the extent of the lesions. Surgical excision remains the cornerstone of management, and histopathological examination remains crucial for a definitive diagnosis. This case highlights the importance of integrating clinical, radiological, and pathological findings to guide therapeutic strategies effectively.

## References

[1] Neumann HP, Wiestler OD (1991). Clustering of features of von Hippel-Lindau syndrome: evidence for a complex genetic locus. Lancet.

[2] Farida L, Kalman T, James G (1993). ​​Identification of the von Hippel-Lindau disease tumor suppressor gene. Science.

[3] Wait SD, Vortmeyer AO, Lonser RR (2004). Somatic mutations in VHL germline deletion kindred correlate with mild phenotype. Ann Neurol.

[4] Maher ER, Iselius L, Yates JR (1991). Von Hippel-Lindau disease: a genetic study. J Med Genet.

[5] Lonser RR, Glenn GM, Walther M, Chew EY, Libutti SK, Linehan WM, Oldfield EH (2003). Von Hippel-Lindau disease. Lancet.

[6] Richard S, Campello C, Taillandier L, Parker F, Resche F (1998). Haemangioblastoma of the central nervous system in von Hippel-Lindau disease. J Intern Med.

[7] Lamiell JM, Salazar FG, Hsia YE (1989). von Hippel-Lindau disease affecting 43 members of a single kindred. Medicine.

[8] Maher ER, Neumann HP, Richard S (2011). von Hippel-Lindau disease: a clinical and scientific review. Eur J Hum Genet.

[9] Walther MM, Reiter R, Keiser HR (1999). Clinical and genetic characterization of pheochromocytoma in von Hippel-Lindau families: comparison with sporadic pheochromocytoma gives insight into natural history of pheochromocytoma. J Urol.

[10] Browne TR, Adams RD, Roberson GH (1976). Hemangioblastoma of the spinal cord. Review and report of five cases. Arch Neurol.

[11] Wanebo JE, Lonser RR, Glenn GM, Oldfield EH (2003). The natural history of hemangioblastomas of the central nervous system in patients with von Hippel-Lindau disease. J Neurosurg.

[12] Jagannathan J, Lonser RR, Smith R, DeVroom HL, Oldfield EH (2008). Surgical management of cerebellar hemangioblastomas in patients with von Hippel-Lindau disease. J Neurosurg.

[13] Ammerman JM, Lonser RR, Dambrosia J, Butman JA, Oldfield EH (2006). Long-term natural history of hemangioblastomas in patients with von Hippel-Lindau disease: implications for treatment. J Neurosurg.

[14] Lonser RR, Weil RJ, Wanebo JE, DeVroom HL, Oldfield EH (2003). Surgical management of spinal cord hemangioblastomas in patients with von Hippel-Lindau disease. J Neurosurg.

[15] Weil RJ, Lonser RR, DeVroom HL, Wanebo JE, Oldfield EH (2003). Surgical management of brainstem hemangioblastomas in patients with von Hippel-Lindau disease. J Neurosurg.

